# Allelopathic Effect of a Chilean Strain of *Karenia selliformis* (Gymnodiniales, Dinoflagellata) on Phytoplankton Species

**DOI:** 10.3390/microorganisms12091834

**Published:** 2024-09-05

**Authors:** Victoria Alfaro-Ahumada, Sandra Jara-Toro, Catharina Alves-de-Souza, Alejandra Rivera-Latorre, Jorge I. Mardones, Juan José Gallardo-Rodriguez, Allisson Astuya-Villalón

**Affiliations:** 1 Laboratorio de Biotoxinas Marinas (LBTx-UdeC), Departamento de Oceanografía, Facultad de Ciencias Naturales y Oceanográficas, Universidad de Concepción, Concepción 4030000, Chile; vialfaro@udec.cl (V.A.-A.); sjara@udec.cl (S.J.-T.); alejandrarivera@udec.cl (A.R.-L.); 2 Centro de Investigación Oceanográfica COPAS Coastal, Universidad de Concepción, Concepción 4030000, Chile; 3 Centro de Estudios de Algas Nocivas (CREAN), Instituto de Fomento Pesquero (IFOP), Puerto Montt 5501679, Chile; jorge.mardones@ifop.cl; 4 Centro de Investigación en Recursos Naturales y Sustentabilidad (CIRENYS), Universidad Bernardo O’Higgins, Santiago 8370993, Chile; 5 Department of Chemical Engineering, Research Centre CIAIMBITAL, University of Almería, 04120 Almería, Spain; jgr285@ual.es

**Keywords:** bloom-forming microalgae, allelopathy, *Karenia selliformis*

## Abstract

Blooms of the dinoflagellate *Karenia selliformis* in Chile, often associated with massive fish kills, have been noted alongside other species from the Kareniaceae family, such as *Karenia* spp. and *Karlodinium* spp. However, the potential allelopathy impact of Chilean *K. selliformis* on other phytoplankton species remains unexplored. Here, we assessed the allelopathic effects of cell-free exudates from a Chilean *K. selliformis* strain on six phytoplankton strains representing diverse microalgal groups. The findings of these experiments offer valuable insights into the varied responses of both non-toxic and toxic microalgae to allelochemicals produced by a toxic microalga, showcasing the intricate and multifaceted nature of allelopathic interactions in microalgal communities. The study revealed species-dependent effects, with variable response in cell growth, photosynthetic efficiency (i.e., *F_v_*/*F_m_*), and intracellular reactive oxygen species (ROS) production. While certain strains exhibited significant growth inhibition in response to the allelochemicals, others demonstrated no apparent effect on cell proliferation, indicating varying sensitivity to specific allelochemicals or potentially distinct detoxification mechanisms. Similarly, the diverse effects on *F_v_*/*F_m_* highlight the complexity of allelopathic interactions, with some species showing reduced efficiency without alterations in intracellular ROS production, while others displayed increased ROS production alongside impaired photosynthesis.

## 1. Introduction

Harmful algal blooms (HABs) are natural events characterized by the proliferation of certain phytoplankton species, which sometimes lead to water discoloration [1]. During HAB events, numerous metabolites produced by the blooming microalgae are released into the water [1,2]. Studies on the effect of these bioactive compounds have been traditionally focused on their toxic effect on humans or animals, either directly or through the trophic web, causing a range of health issues from mild to severe, and in the most severe cases, death [3,4]. However, there is limited understanding of the impact of microalgal secondary metabolites on plankton assemblages.

One of the main open questions in phytoplankton ecology is the reason behind the formation of blooms by certain microalgae. In general, species that can effectively outcompete with others for nutrients are expected to dominate phytoplankton assemblages. However, HABs often consist of dinoflagellates, which, due to their slower growth and nutrient uptake rates [5], are considered less competitive compared to other phytoplankton groups like diatoms [6]. It has been proposed that the main ecological function of producing “toxic” secondary compounds by some HAB species is to hinder the growth of competing phytoplankton species and/or to decrease cell losses by deterring their grazers [7].

Allelopathy is defined as the direct or indirect interactions facilitated by the release of secondary metabolites (i.e., allelochemicals) by some organisms, including plants, algae, bacteria, and fungi, which exhibit an inhibitory effect on the germination, growth, survival, and reproduction of other organisms [8,9]. Growing observational and experimental evidence indicates that allelopathic interactions play a crucial role in mediating phytoplankton species succession and the proliferation of HAB species. By inhibiting the growth and competitiveness of specific organisms, allelochemicals can create favorable conditions for the proliferation of species that are resistant to those compounds. This selective suppression of competing species through allelopathic interactions has the potential to drive shifts in community structure [10,11,12,13]. Common microalgal allelochemical-induced effects include inhibition of growth and photosynthesis inhibition, as well as the death of the target cells [14,15]. However, the mechanisms triggering microalgae allelopathy are not yet well understood [16]. Examples include the inhibitory impact of metabolites released by the raphidophytes *Heterosigma akashiwo* Hada ex Hara & Chihara and *Chattonella antiqua* (Hada) Ono on the growth of the diatom *Skeletonema costatum* (Greville) Cleve [17], and the oxidative effects induced by *Karenia mikimotoi* (Miyake & Kominami ex Oda) Gert Hansen & Moestrup on the diatom *Thalassiosira pseudonana* Hasle & Heimdal, leading to decreased photosynthesis efficiency and nutrient uptake [15]. Blooms of the toxic dinoflagellate *Karenia selliformis* A.J.Haywood, K.A. Steidinger & L. MacKenzie are among the most harmful recorded HAB events due to their production of biotoxins, ichthyotoxins, and allelochemicals that have adverse effects on other phytoplankton species, trophic food webs, and wildlife populations such as birds, mammals, and fish [18]. In Chile, these blooms often coincide with other species from the family Kareniaceae (i.e., *Karenia* spp. and *Karlodinium* spp.), making it challenging to isolate the specific environmental effects of *K. selliformis* toxins. However, recent characterization of two *K. selliformis* strains isolated during a large bloom of Kareniaceae species in southern Chilean Patagonia in 2018 revealed intriguing findings [19]. Notably, the absence of gymnodimines (GYMs), typically associated with HAB events of Kareniaceae species in different coastal regions, was observed. Instead, there was a high presence of potentially harmful polyunsaturated fatty acids (PUFAs) and the identification of two compounds with similar mass transition to brevenal, a brevetoxin-related non-toxic compound [20]. In vitro assays demonstrated the high cytotoxicity of the two strains of *K. selliformis* on the rainbow trout gill RTgill-W1 cell line, suggesting significant potential for fish mortality. However, the potential allelopathic effect of these secondary metabolites remains to be investigated.

In this study, we assessed the allelopathic impact of one of the two Chilean *K. selliformis* strains isolated during the 2018 Kareniaceae bloom on various phytoplankton species. The bioassays involved exposing the cell-free exudate of *K. selliformis* (strain CREAN-KS02 isolated from the Aysén region at 43° S) to the target microalgae *Phaeodactylum tricornutum* Bohlin UCN-B018-1, *Thalassiossira pseudonana* UCN-B011-2, *Rhodomonas salina* (Wisłouch) D.R.A. Hill & R. Wetherbee CS-174, and *Dunaliella tertiolecta* Butcher UTEX-999, as well as two strains of *H. akashiwo* isolated from Chilean Patagonia (CREAN HA-01) and New Zealand (CCMP302). Effects on cell density, photosynthetic efficiency, production of reactive oxygen species (ROS), and morphology of the exposed cells were evaluated.

## 2. Materials and Methods

### 2.1. Microalgae Culture

The *K. selliformis* (strain CREAN-KS02), isolated from the Chilean Aysen region [19], was obtained from the Collection of Harmful Algae hosted at the Center for Harmful Algae Studies at the Chilean Institute for Fishery Development (CREAN/IFOP; Puerto Montt, Chile) and subsequently maintained at the Laboratory of Marine Biotoxins of the University of Concepción (LBTx-UdeC; Concepción, Chile). The strains of the six microalgal species used as targets were obtained from different sources. The chlorophyte *D. tertiolecta* (UTEX-999; CCM-UDEC-75) and the cryptophyte *R. salina* (CS-174/CCM-UDEC-153), isolated from the Oslofjord (Norway) and Milford (CT, USA), respectively, were provided by the Laboratory of Phycology of the University of Concepción (FICOLAB-UdeC). The diatoms *P. tricornutum* (strain UCN-B018-1) and *T. pseudonana* (strain UCN-B011-2), with unknown geographical origins, were donated by Dr. Alvarez-Vergara from the Department of Aquaculture at the Northern Catholic University (Antofagasta, Chile). Additionally, two strains of the raphidophyte *H. akashiwo*, strain CCMP302 from New Zealand and strain CREAN-HA01 from the Chilean Los Lagos region, were utilized. All strains were cultured in L1 medium (supplemented with sodium silicate for diatoms [21], prepared with seawater from the coastal area off central Chile (36°31′ S, 73°08′ W), 0.2 µm filtered, and autoclaved, with salinity previously adjusted to 32 PSU (the salinity at which blooms of *K. selliformis* CREAN-KS02 have been observed in the field [22]). Cultures were maintained in medium L1 at 17 °C, under irradiance of 100 μmol photon m^−2^ s^−1^ and a 16 h:8 h light:dark photoperiod (except for *K. selliformis* and *H. akashiwo* strains, which were grown at 140 μmol photon m^−2^ s^−1^).

Growth curves *of K. selliformis* CREAN-KS02 were determined from 500 mL cultures started at 1000 cells mL^−1^, inoculated from stock cultures in the exponential phase. Daily cell densities were determined using Sedgewick Rafter chambers [23]. Growth curves of the target phytoplankton species were derived from 50 mL cultures initiated at 20,000 cells mL^−1^ from stock cultures at the exponential phase, with daily cell counts obtained with Neubauer chambers [23]. For both *K. selliformis* and target phytoplankton, cell growth rates (i.e., divisions per day) were determined during the exponential growth phase [24] according to the following equation:

$$\mu^{-1}=\frac{1}{t_{2}-t_{1}}\times log\left(\frac{N_{2}}{N_{1}}\right)$$

where *µ*^−1^ is the growth rate, *n*_1_ is the initial cell concentration, *n*_2_ is the final cell concentration, *t*_1_ is the initial time, and *t*_2_ is the final time.

### 2.2. Experimental Setup

Exudates from *K. selliformis* CREAN-KS02 were collected in triplicate from cultures in the exponential growth phase (9000–11,000 cells mL^−1^) by gentle vacuum filtration (<300 mm Hg) through a 0.47 µm glass fiber filter (Merck, Millipore, Dublin, Ireland). To prevent the potential absorption of secondary metabolites by plastic materials [25], the obtained cell-free exudates were gathered into glass tubes at room temperature and promptly used to avoid degradation of potential allelopathic compounds. Inoculations were carried out in triplicates using 100 mL glass Erlenmeyer flasks, where 20 mL of fresh cell-free exudate was added to cultures of the various target phytoplankton species (final concentration was ~20,000 cells mL^−1^) and topped with L1 media to reach a final volume of 50 mL. The control cultures of the target phytoplankton species (also in triplicate) received only fresh L1 medium without the addition of exudate.

### 2.3. Allelopathic Effect on Phytoplankton Growth

The maximal growth rates of the target phytoplankton species were determined based on cell counts obtained at 0 h, 12 h, 24 h, and 48 h, following the methodology previously outlined. Additionally, microphotographs were taken 12 h post-exposure using a Nikon Eclipse Ti E200 epifluorescence microscope (Tokyo, Japan) with a Euromex MOD DC.6000I camera (Duiven, The Netherlands). Additionally, microphotographs were taken 12 h post-exposure using the Eclipse Ti E200 and the Euromex MOD DC.6000I.

### 2.4. Photosynthetic Efficiency (F_v_/F_m_)

Samples (2 mL) of the target phytoplankton strains from all replicates were collected on day 0 immediately after inoculation and acclimated to darkness for 15 min before assessing their photochemical performance (every 15 min for 105 min) using pulse amplitude modulation fluorometry (PAM) with a portable AquaPen-C AP-C 100 (Photon Systems Instruments, Drassov, Czech Republic). Then, the photosynthetic efficiency (*F_v_*/*F_m_*; [26]) was estimated using the following formula:

$$\frac{F_{v}}{F_{m}}=\frac{\left(F_{m}-F_{0}\right)}{F_{m}}$$

where *F*_0_ is the initial fluorescence intensity, *F_m_* is the maximum intensity under saturated light conditions (0.5 s at 1500 mmol photon m^−2^ s^−1^), and *F_v_* is 1/4 of (*F_m_* − *F*_0_).

### 2.5. Reactive Oxygen Species (ROS)

The relative levels of reactive oxygen species (ROS) were determined using 2′,7′dichlorodihydrofluorescein diacetate (H_2_DCFDA; Invitrogen, San Diego, CA, USA), which, in the presence of ROS, is converted into the highly fluorescent compound 2′,7′dichlorofluorescein (DCF). This method has previously been used to measure intracellular ROS production in dinoflagellates [5,27]. In this process, duplicate samples (190 µL) taken from each replicate at 0 h were inoculated with 10 µL of H_2_DCFDA and the emitted fluorescence was measured every 30 min for 12 h using a Synergy H1 microplate reader (BioTek, Winooski, VT, USA), with excitation of 485/20 nm and emission at 528/20 nm. DCF fluorescence data were expressed as absolute fluorescent units, and background fluorescence 0 h was subtracted from each measurement.

### 2.6. Statistical Analysis

Growth, photosynthetic activity, and ROS production in the six phytoplankton strains exposed to *K. selliformis* CREAN-KS02 cell-free exudate were compared to controls (i.e., cultures not exposed to exudate) by *t* tests using the *t.test* function in basic package of R software (https://cran.r-project.org/ (accessed on 20 March 2024)). Prior to analysis, the within-group normality in each experimental treatment and controls was checked using the Shapiro–Wilk test (function *shapiro_test* in R basic package), while the homogeneity of variance was checked with Levene’s test (*leveneTest* function in ‘car’ package).

## 3. Results

### 3.1. Allelopathic Effect of K. selliformis on Phytoplankton Growth

**Figure 1:**
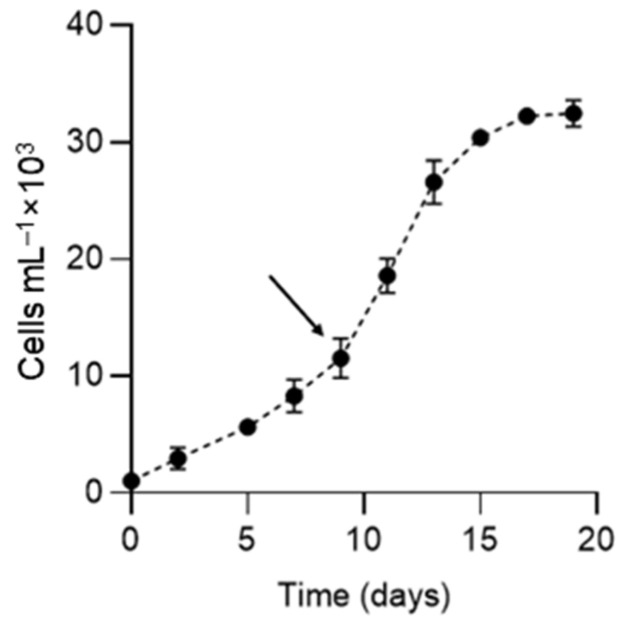
Growth curve of *Karenia selliformis* CREAN-KS02. The arrow shows the time at which the cell-free exudate was obtained. Data are presented as means ± SD (n = 3).

**Figure 2:**
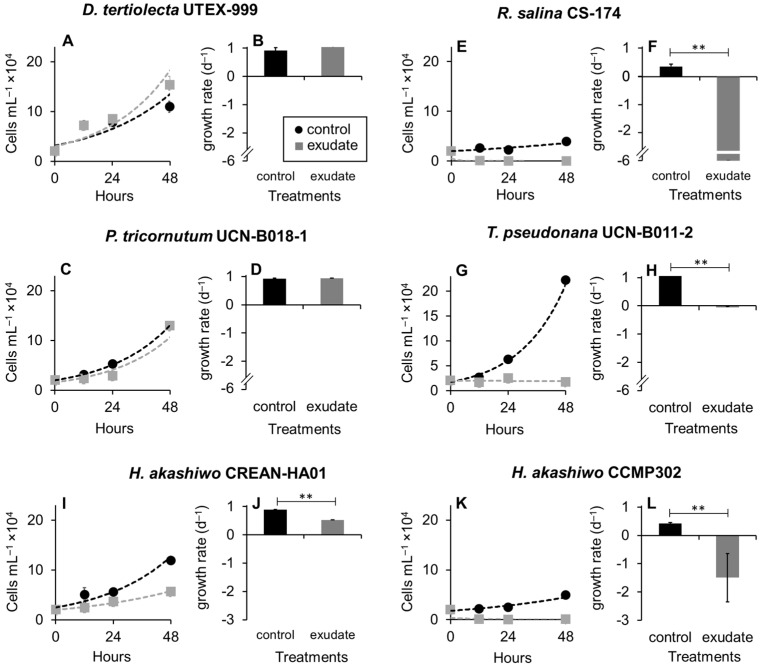
Effect of *Karenia selliformis* CREAN-KS02 cell-free exudate on the cell concentration (**A**,**C**,**E**,**G**,**I**,**L**) and growth rates (**B**,**D**,**F**,**H**,**J**,**L**) of the six target phytoplankton species over 48 h of exposure: (**A**,**B**) *Dunaliella tertiolecta* UTEX-999, (**C**,**D**) *Rhodomonas salina* CS-174, (**E**,**F**) *Phaeodactylum tricornutum* UCN-B018-1, (**G**,**H**) *Thalassiosira pseudonana* UCN-B011-2, (**I**,**J**) *Heterosigma akashiwo* CREAN-HA01, (K,**L**) *H. akashiwo* CCMP302. The data are shown as means ± SD (n = 3), ** *p* > 0.01.

**Figure 3:**
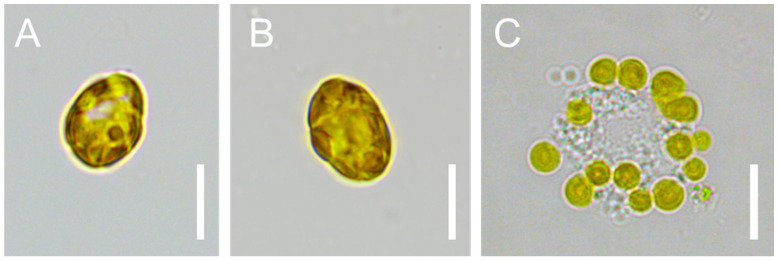
Cells of *Heterosigma akashiwo* CCPM302 in control (**A**,**B**) and exposed (**C**) to *Karenia sellliformis* CREAN-HA01 cell-free exudate. Scale bars = 10 µm.

*K. selliformis* CREAN-KS02 cultures were typically grown to reach cell densities of 35,000 cells mL^−1^, with the exponential phase lasting from day 5 to 13 (Figure 1). Cell-free exudate was collected from cells during the mid-exponential phase on day 9. When exposed to *K. selliformis* cell-free exudate, *D. tertiolecta* UTEX-999 did not show any negative effects, as its cell concentrations remained consistent with those of the control throughout the experiment (Figure 2A,B). On the other hand, *R. salina* CS-174 experienced a drastic lethal effect upon exposure to *K. selliformis* CREAN-KS02, with 100% mortality observed shortly (12 h) after inoculation (Figure 2C,D). Contrasting results were observed for the two tested diatoms: while *P. tricornutum* UCN-B018-1 did not exhibit any adverse effect (Figure 2E,F), a significant inhibitory effect was observed in *T. pseudonana* UCN-B011-2 12–24 h post-inoculation (Figure 2G,H). *H. akashiwo* CREAN-HA01 from Chilean Patagonia displayed considerable susceptibility to *K. selliformis* cell-free exudate (~50% mortality) after 48 h of exposure (Figure 2I,J). However, this effect was less pronounced compared to the response of *H. akashiwo* CCPM302 from New Zealand, which exhibited a substantial decrease in cell concentration compared to the control cultures (>90%) 12 h after inoculation (Figure 2K,L). Additionally, abnormal cell morphology was observed by optical microscopy (Figure 3).

### 3.2. Photosynthetic Efficiency (F_v_/F_m_)

**Figure 4:**
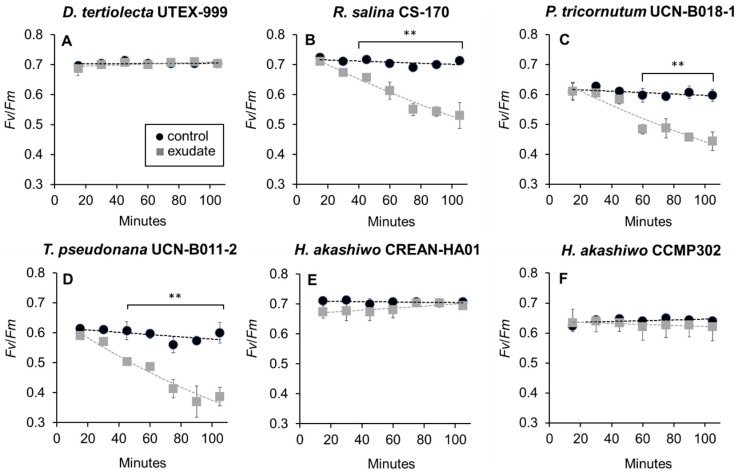
Photosystem II maximum quantum performance (*F_v_*/*F_m_*) of the phytoplankton species exposed to *Karenia selliformis* CREAN-KS02 cell-free exudate compared to control cultures: (**A**) *Dunaliella tertiolecta* UTEX-999, (**B**) *Rhodomonas salina* CS-174, (**C**) *Phaeodactylum tricornutum* UCN-B018-1, (**D**) *Thalassiosira pseudonana* UCN-B011-2, (**E**) *Heterosigma akashiwo* CREAN-HA01, (**F**) *H. akashiwo* CCMP302. The data are shown as means ± SD (n = 3), ** *p* < 0.01.

Under control conditions, the quantum yields (*F_v_*/*F_m_*) for the six phytoplankton strains were between 0.6 and 0.7 (Figure 4). The addition of *K. selliformis* CREAN-KS02 cell-free exudate affected the *F_v_*/*F_m_* values of *D. tertiolecta* UTEX-999 (Figure 4A), while sustained decrease in this parameter was observed in *R. salina* CS-174 (Figure 4B), *P. tricornutum* UCN-B018-1 (Figure 4C), and *T. pseudonana* UCN-B011-2 (Figure 4D) within the initial 120 min of exposure. Conversely, no significant effect was observed on the *F_v_*/*F_m_* values of *H. akashiwo* CCMP302 (Figure 4E) and *H. akashiwo* CREAN-HA01 (Figure 4F) in comparison to control cultures.

### 3.3. ROS Production

**Figure 5:**
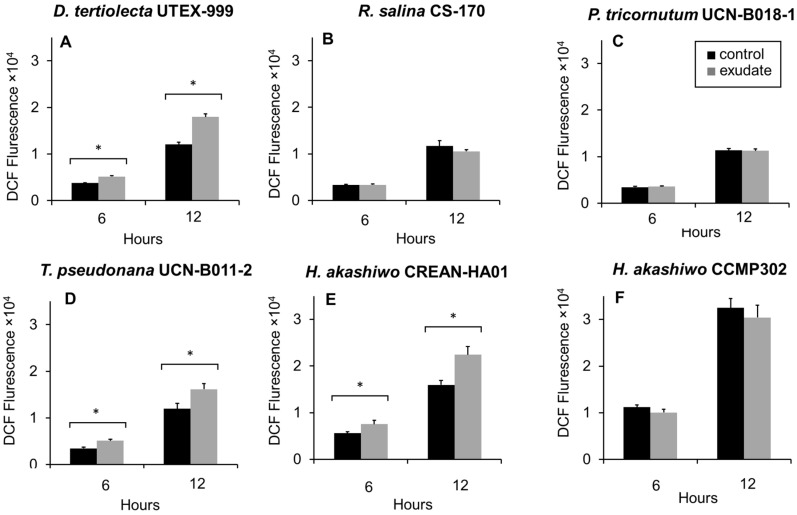
Fluorescence of 2′,7′dichlorofluorescein (DCF) associated with intracellular reactive oxygen species (ROS) production in the target phytoplankton strains after 6 h and 12 h of exposure to *Karenia selliformis* CREAN-KS02 cell-free exudate: (**A**) *Dunaliella tertiolecta* UTEX-999, (**B**) *Rhodomonas salina* CS-174, (**C**) *Phaeodactylum tricornutum* UCN-B018-1, (**D**) *Thalassiosira pseudonana* UCN-B011-2, (**E**) *Heterosigma akashiwo* CREAN-HA01, (**F**) *H. akashiwo* CCMP302. The data are shown as means ± SD (n = 3), * *p* < 0.05.

The intracellular levels of ROS were assessed using the fluorescent DCF probe after 6 h and 12 h of exposure to *K. selliformis* CREAN-KS02 cell-free exudates (Figure 5). In general, DCF fluorescence was distributed in the whole area of the microalgal cells under a fluorescence microscopen. Following 6 h and 12 h of exposure, a significant increase in DCF fluorescence was detected in *D. tertiolecta* UTEX-999 (Figure 5A), *T. pseudonana* UCN-B011-2 (Figure 5D)*,* and *H. akashiwo* CREAN-HA01 (Figure 5E). Conversely, there were no significant differences observed in DCF fluorescence levels in *R. salina* CS-174 (Figure 5B), *P. tricornutum* UCN-B018-1 (Figure 5C), or *H. akashiwo* CCMP302 (Figure 5F) compared to the control cultures.

## 4. Discussion

Blooms of *K. selliformis* in Chile have often coincided with other species of the family Kareniaceae [28,29,30]). While the toxin profile of Chilean *K. selliformis* strains has been only partially characterized [19], their high cytotoxicity has been confirmed through in vitro bioassays based on the RTgill-W1 cell line [30,31]. However, the potential allelopathic effect of Chilean *K. selliformis* is yet to be evaluated. Plankton allelopathy is typically studied in laboratory-controlled experiments using target microalgal species (e.g., [12,32,33]. For that, it is crucial to carefully select the target organism and employ a range of methods (proxies) to elucidate the inhibition mechanisms fully or partially. In this study, we conducted the first assessment of the effects of exudates from a Chilean *K. selliformis* strain (CREAN-KS02) on cell growth, photosynthetic efficiency (i.e., *F_v_*/*F_m_*), and intracellular ROS production of six phytoplankton microalgal strains from various taxonomic groups. Our findings demonstrate that the allelopathic effect of Chilean *K. selliformis* CREAN-KS02 was not only species-dependent but also exhibited variability among strains within the same target phytoplankton species.

In this study, all target phytoplankton species exhibited adverse effects from the cell-free exudates derived from *K. selliformis* CREAN-KS02, although high variability was observed in both the strength and nature of the allelopathic impact (Figure 3, Figure 4 and Figure 5, Table 1). The allelochemical compounds originating from microalgae encompass a wide array of metabolites with diverse chemical properties (e.g., PUFAS, ROS, and phycotoxins) that can influence phytoplankton species through various mechanisms, including cell membrane damage by lytic activity, inhibition of photosynthesis and enzymatic activity, increased oxidative stress, apoptosis, and effects on cell mobility [34,35,36,37]. Such a diversity in toxic mechanisms is expected to cause diverse effects on target phytoplankton species from different microalgal groups.

Cell growth inhibition was observed in *R. salina* CS-174, *T. pseudonana* UCN-B011-2, and the two *H. akashiwo* strains (CREAN-HA01 and CCMP302). Among the impacted strains, *T. pseudonana* UCN-B011-2 and *R. salina* CS-174 displayed a significant reduction in growth (Figure 3B and Figure 3C, respectively), with *R. salina* CS-174 experiencing a more severe decline, reaching zero cell densities just 12 h post-exposure. Additionally, both strains exhibited decreased *F_v_*/*F_m_* values (Figure 4B and Figure 4C, respectively), while *T. pseudonana* UCN-B011-2 showed increased intracellular ROS production (Figure 5C). This prominent susceptibility to allelopathic compounds aligns with previous studies using strains of these two species as target organisms [14,15,38,39,40], with *R. salina* strains being frequently used as model species in lithic bioassays for the screening of allelopathic activities in microalgae [11,32].

The growth of the two *Heterosigma akashiwo* strains was negatively affected by *K. selliformis* CREAN-KS02 cell-free exudate (Figure 4D,E). However, strain CCMP302 (from New Zealand) exhibited a substantially higher mortality rate of 90% compared to strain CREA-HA01, which displayed a 50% reduction in growth compared to control replicates. While the photosynthetic efficiency (*F_v_*/*F_m_*) remained unaffected in both strains, only CREA-HA01 showed an increased intracellular ROS production upon exposure to *K. selliformis* CREAN-KS02 cell-free exudate. It is noteworthy that the two tested *H. akashiwo* strains originated from distinct geographical regions, with the Chilean strain CREAN-HA01, isolated from the same location where *K. selliformis* blooms have also been reported, demonstrating greater resistance to the allopathic effect of strain *K. selliformis* CREAN-KS02. This observation may suggest a sympatric adaptation of the Chilean *H. akashiwo* strain to the allelopathic compounds produced by *K. selliformis*. This underscores the importance of gaining a deeper understanding of the specific impacts of toxic microalgal exudates on various local phytoplankton species, which could have ecological implications for aquatic ecosystems.

Despite *D. tertiolecta* UTEX-999 and *P. tricornutum* UCN-B018-1 not exhibiting growth inhibition when exposed to *K. selliformis* CREAN-KS02 cell-free exudates (Figure 3A and Figure 3C, respectively), both strains were affected to some extent, with *D. tertiolecta* experiencing a decrease in photosynthetic efficiency (Figure 4A) and *P. tricornutum* showing an increase in intracellular ROS production (Figure 5C). These findings differ from previous allelopathy studies using *D. tertiolecta* as the target species, which reported negative impacts of polyunsaturated aldehydes (PUAs) on cell growth and cell membrane integrity [41]. Similarly, previous research has indicated that okadaic acid (OA) can impair growth and photosynthetic electron transport, leading to increased intracellular ROS production in *D. tertiolecta* [42]. Moreover, inhibitory effects on cell division and chloroplast damage have been reported in *P. tricornutum* when co-cultured with *Alexandrium tamarense* [43], while domoic acid (DA) has been shown to increase intracellular ROS production in this species [44].

Photosynthetic efficiency is a widely used indicator of microalgal cell health in both field populations and cultures [45]. Here, *F_v_*/*F_m_* values in all control replicates fell between 0.65 and 0.7, a range typically associated with photosynthetically healthy cultures [46], indicating that the six microalgal strains were in a photosynthetically efficient state prior to exudate exposure. Allelopathic compounds have the potential to disrupt photosynthetic machinery by affecting processes like chlorophyll synthesis or electron transport chain activity, resulting in decreased efficiency in converting light into chemical energy [47]. These compounds can trigger stress responses in microalgae, leading to reduced photosynthetic rates as cells allocate resources to cope with the stressor instead of investing in growth and reproduction [48]. Decreased photosynthetic efficiency can thus be used as a proxy for assessing the allelopathic effect of compounds on microalgae, with changes in efficiency reflecting cellular stress and physiological impairment [12,33]. For instance, reports have shown that the diatom *T. pseudonana* experienced allelopathically effects through inhibition of its photosynthetic apparatus when exposed to exudates from the dinoflagellates *Alexandrium pacificum* [14] and *Karenia brevis* [15]. This highlights that incorporating methods like pulse amplitude fluorometry (PAM) in studies of allelopathic effects is a valuable and easily implementable methodological strategy to include in this type of ecological toxicity study [12].

Allelopathic compounds can trigger oxidative stress in microalgae by disrupting the cellular redox balance, resulting in an increased production of intracellular ROS (e.g., O_2_^−^, H_2_O_2_) as a defense mechanism [48,49]. This spike in intracellular ROS levels can subsequently trigger apoptosis pathways [50,51] and/or damage the photosynthetic apparatus [47,48]. The continued increase in ROS production observed up to 12 h of exposure suggests a lasting impact of allelopathic compounds on the target species, a phenomenon reported for other microalgal species exposed to HAB-producing ichthyotoxic microalgae, such as species of the raphidophytes genera *Heterosigma* and *Chatonella* [52,53,54,55].

The absence of increased intracellular ROS production in *R. salina* CS-174 and the two *H. akashiwo* strains CREA-HA01 and CCMP302, despite exhibiting high cell mortality when exposed to *K. selliformis* cell-free exudate, indicates a complex interplay of factors influencing cellular responses to allelopathic compounds, with several explanations accounting for this observation. One possibility is that exposure to allelochemicals may trigger rapid extensive cell death, resulting in the cessation of metabolic activity or cell lysis before a significant accumulation of ROS occurs [56]. Alternatively, the primary damage in *R. salina* could be lytic, as described by other researchers [32].

While it is typically expected that decreased photosynthetic efficiency would be correlated with increased ROS production [5], the findings from this study revealed some unique patterns. Among the tested strains, only *T. pseudonana* UCN-B011-2 displayed both decreased *F_v_*/*F_m_* and increased intracellular ROS production (Figure 3D and Figure 4D; Table 1). The elevated intracellular ROS production observed in *P. tricornutum* UNC-B018-1 suggests that this strain might have experienced oxidative stress early in the experiment, resulting in ROS generation either as a defense mechanism or due to cellular damage induced by allelochemicals. In contrast, *D. tertiolecta* UTEX-999 exhibited decreased *F_v_*/*F_m_* levels without a concomitant increase in intracellular ROS production. This discrepancy could be explained by the timing of measurements, since *F_v_*/*F_m_* was measured at the onset of the experiment (0 h), likely capturing the immediate impact of allelopathic compounds on photosynthetic processes. Meanwhile, intracellular ROS production was evaluated at later times (6 h and 12 h), allowing for the detection of ROS-related responses that may have emerged after the initial assessment of photosynthetic efficiency.

The findings of this study indicate that Chilean *K. selliformis* populations produce allelochemicals, with the allelopathic effect being specific to each target species. Interestingly, the two *H. akashiwo* strains exhibited contrasting responses, with the strain isolated from the same geographic region as *K. selliformis* demonstrating greater resistance to its allelopathic effect. The observed discrepancies, such as changes in *F_v_*/*F_m_* or intracellular ROS production without concomitant alterations in cell growth, underscore the intricate nature of allelopathic interactions and the varied cellular responses to allelochemicals. These differences could signify varying sensitivities to specific allelochemicals or variability in the activation of defense mechanisms across microalgal species. Moreover, the timing of allelochemical exposure and cellular responses may play a role in the observed variability, emphasizing the need for further studies to unravel the mechanisms driving species-specific responses to allelopathy in microalgae.

## 5. Conclusions

Our results underscore the significance of ongoing research on *K. selliformis* toxicity and its ecological effects on marine ecosystems in Chile, particularly in understanding the impact of its allelopathic effects on phytoplankton ecology and harmful algal bloom dynamics in the Chilean fjords. Documented allelopathic interactions shed light on how allelochemicals can affect various microalgal species, influencing factors such as cell growth, photosynthetic efficiency, and intracellular ROS production. Future investigations should delve deeper into the specific allelochemical mechanisms at play and their effects on different target species. Furthermore, exploring the adaptability of local microalgal populations, such as the Chilean *H. akashiwo* strain, to allelopathic compounds produced by *K. selliformis* could offer insights into local ecological dynamics and potential adaptations. By further exploring these allelopathic effects, researchers can enhance their understanding of how these interactions shape phytoplankton communities and potentially impact marine ecosystems in the Chilean fjords.

## Figures and Tables

**Table 1:** Summary of allelopathic effects of *K. selliformis* CREAN-KS02 cell-free extract on growth, photosynthetic efficiency (*F_v_*/*F_m_*) and ROS production of the six tested target phytoplankton strains. Positive (+) and negative (−) signs indicate significant (*p* < 0.05) and insignificant differences from controls, respectively.

Target Strain	Geographical Origin	Allelopathic Effect		
		Growth	*F_v_*/*F_m_*	ROS
*Dunaliella tertiolecta* UTEX-999	Oslo fjord, Norway	−	−	+
*Rhodomonas salina* CS-174	Milford, USA	+	+	−
*Phaeodactilum tricornutum* UNC-B018-1	Unknow	−	+	−
*Thalassiosira pseudonana* UNC-B011-2	Unknow	+	+	+
*Heterosigma akashiwo* CREAN-HA01	Patagonian fjord, Chile	+	−	+
*Heterosigma akashiwo* CCMP302	New Zealand	+	−	−

## Data Availability

The original contributions presented in the study are included in the article, further inquiries can be directed to the corresponding authors.
